# The Anti-Oxidative and Anti-Neuroinflammatory Effects of *Sargassum horneri* by Heme Oxygenase-1 Induction in BV2 and HT22 Cells

**DOI:** 10.3390/antiox10060859

**Published:** 2021-05-27

**Authors:** Wonmin Ko, Hwan Lee, Nayeon Kim, Hee Geun Jo, Eun-Rhan Woo, Kyounghoon Lee, Young Seok Han, Sang Rul Park, Ginnae Ahn, Sun Hee Cheong, Dong-Sung Lee

**Affiliations:** 1 Department of Marine Bio-Food Sciences, Chonnam National University, Yeosu 59626, Korea; rabis815@naver.com (W.K.); altkwh@naver.com (H.G.J.); gnahn@chonnam.ac.kr (G.A.); 2 College of Pharmacy, Chosun University, Dong-gu, Gwangju 61452, Korea; ghksdldi123@hanmail.net (H.L.); rlaskdus1209@naver.com (N.K.); wooer@Chosun.ac.kr (E.-R.W.); 3 Division of Fisheries Science, Chonnam National University, Yeosu 59626, Korea; ricky1106@naver.com; 4 Neo Environmental Business Co., Daewoo Technopark, Doyak-ro, Bucheon 14523, Korea; hanulva@neoenbiz.com; 5 Estuarine and Coastal Ecology Laboratory, Department of Marine Life Sciences, Jeju National University, Jeju 63243, Korea; srpark@jejunu.ac.kr

**Keywords:** *Sargassum horneri*, CH_2_Cl_2_-soluble fraction, antioxidant activity, anti-neuroinflammatory effects, heme oxygenase-1, BV2 cells, HT22 cells

## Abstract

*Sargassum horneri* is used as a traditional medicinal agent and exhibits various pharmacological effects. In this study, we found that the 70% EtOH extract contained 34.37 ± 0.75 μg/mg fucosterol. We tested the antioxidant activities of the 70% EtOH extracts and their fractions. The CH_2_Cl_2_-soluble fraction showed the strongest DPPH and ABTS radical scavenging activities. Next, we evaluated the anti-neuroinflammatory effects of *S. horneri* on lipopolysaccharide (LPS)-stimulated BV2 cells. Pretreatment with the extract and fractions suppressed LPS-induced production of nitric oxide (NO) in BV2 cells. The 70% EtOH, CH_2_Cl_2_-soluble fraction, and water-soluble fraction inhibited the production of prostaglandin E2, interleukin-6, and tumor necrosis factor-α, as well as markedly blocking LPS-induced expression of inducible NO synthase and cyclooxygenase-2 via inactivation of the nuclear factor-kappa B pathway. In addition, the CH_2_Cl_2_-soluble fraction showed the most remarkable heme oxygenase (HO)-1 expression effects and increased nuclear erythroid 2-related factor translocation in the nucleus. In HT22 cells, the CH_2_Cl_2_-soluble fraction inhibited cell damage and ROS production caused by glutamate via the regulation of HO-1. Therefore, CH_2_Cl_2_-soluble fractions of *S. horneri* can attenuate oxidative action and neuroinflammatory responses via HO-1 induction, demonstrating their potential in the treatment of neuroinflammatory diseases.

## 1. Introduction

Oxidative stress is known to be an important cause of aging and various nervous system disorders. It is biologically produced by oxidative imbalance. Oxidative imbalance occurs due to the formation of reactive oxygen species (ROS) and abnormalities in the antioxidant system of the body [1]. Oxygen plays an important role in maintaining life and is involved in various bodily activities. However, the accumulation of free radicals and ROS can cause acute and chronic diseases. In addition, the accumulation of oxidative stress and ROS is known to intensify neurodegenerative disorders such as Alzheimer’s disease and Parkinson’s disease by causing the destruction and dysfunction of neuronal cells [1,2]. Chronic inflammatory diseases are a significant cause of death, and patients with inflammation-related diseases, including stroke, diabetes mellitus, and ischemic heart disease, have a mortality rate of more than 50% [3]. Therefore, regulation of the immune system and inflammatory processes, which are involved in mental and physical health problems, is an important factor in morbidity and mortality [4,5,6,7]. In neuroinflammation, microglia, which are immune cells residing in the brain, represent the first line of immune defense [8]. These cells are the smallest glial cells in the central nervous system (CNS) and play an essential role in mediating inflammatory responses in the CNS [8]. In response to stimulation with lipopolysaccharide (LPS), microglial cells produce pro-inflammatory mediators such as nitric oxide (NO) and prostaglandin E2 (PGE_2_). The production of these pro-inflammatory mediators is modulated by the inflammatory enzymes, such as inducible NO synthase (iNOS) and cyclooxygenase-2 (COX-2) [8]. Furthermore, pro-inflammatory cytokines, including tumor necrosis factor-α (TNF-α) and interleukins (ILs), are produced by activated microglia [8]. It is well-known that the transcription factor nuclear factor-kappa B (NF-κB) regulates the expression of pro-inflammatory enzymes and cytokines. Under normal conditions, the inhibitor of κB (IκB) protein is coupled with the NF-κB heterodimer present in the cytoplasm [9]. However, various stimuli lead to IκB degradation through the ubiquitin-proteasome system [9]. Consequently, the free NF-κB heterodimer translocates into the nucleus and induces the expression of inflammatory mediators and cytokines. Among the factors regulating oxidative damage and inflammation, phase II enzymes are important. One phase II enzyme, heme oxygenase-1 (HO-1), can metabolize heme to produce carbon monoxide (CO), biliverdin, and iron. These substances are known to have antioxidant and anti-neuroinflammatory actions, and the expression of HO-1 inhibits neuronal cell damage caused by oxidative stress or inflammatory response [10,11].

Species of the genus *Sargassum* are abundant on Jeju Island and in the southern sea of Korea. *Sargassum* seaweed has been listed in ancient Chinese herbal medicine books as a traditional medicinal ingredient [12]. *Sargassum horneri* is a nutrient-rich brown alga and an edible seaweed [13]. *S. horneri* is used as a savory and nutrient-rich sea vegetable in China and Korea [13,14]. *S. horneri* has also been used as a traditional medicinal agent to relieve hypertension, hyperlipidemia, and inflammatory diseases [15]. However, drifting *S. horneri* biomasses, called ‘golden tides’, have accumulated and caused devastating impacts on the coastal ecosystems in recent years along the southern sea of Korea [16]. Thus, the proper handling and application of accumulated *S. horneri* biomass has received increasing attention. Many studies have reported that *Sargassum* spp. exhibit antioxidant activities, such as 2,2-diphenyl-1-picrylhydrazyl (DPPH) scavenging activity, deoxyribose scavenging activity, and hydroxyl radical scavenging activity [17,18,19,20,21]. Moreover, *Sargassum* spp. have also been shown to exhibit anti-cancer and cytotoxic activity [22,23,24]. Extracts of diverse *Sargassum* spp. have been screened for their anti-inflammatory activities [14,25,26]. However, the anti-neuroinflammatory effects of *S. horneri* extracts and their fractions have not yet been reported. In this study, we investigated the antioxidant and anti-neuroinflammatory effects of *S. horneri* extract and its fractions on HO-1 induction in LPS-stimulated BV2 cells. Moreover, the neuroprotective properties of its fractions were also confirmed in HT22 cells.

## 2. Materials and Methods

### 2.1. Preparation of Extract and Sub-Fractions from S. horneri

Samples of *S. horneri* collected off the Jeju coast were donated by Prof. Sang Rul Park (Jeju National University) in April 2019. Dried *S. horneri* (570 g) was extracted with 70% EtOH under reflux, and 112.517 g of residue was produced. Among the total 112.517 g of 70% EtOH, only 70 g of extract was re-suspended in water and then partitioned sequentially with equal volumes of *n-hexane*, dichloromethane (CH_2_Cl_2_), ethyl acetate (EtOAc), and *n*-butanol (*n*-BuOH). Each fraction was evaporated in vacuo to yield the *n-*hexane (6.9 g)-, CH_2_Cl_2_ (8.6 g)-, EtOAc (0.25 g)-, *n*-BuOH (5.1 g)-, and water (47.15 g)-soluble fractions. Each fraction was evaporated in vacuo to finally form a powder, then dissolved in DMSO and used in each experiment.

### 2.2. Materials

Cell culture reagents including fetal bovine serum (FBS) and RPMI-1640 medium were bought from Gibco BRL Co. (Grand Island, NY, USA). Primary antibodies against p65, COX-2, iNOS, HO-1, PCNA, and β-actin were obtained from Santa Cruz Biotechnology (Santa Cruz, CA, USA). Anti-mouse, anti-rabbit, and anti-goat secondary antibodies were bought from Millipore (Billerica, MA, USA). The enzyme-linked immunosorbent assay (ELISA) kits for IL-6, TNF-α, and PGE_2_ were bought from R&D Systems (Minneapolis, MN, USA). All other chemicals were bought from Sigma-Aldrich (St. Louis, MO, USA).

### 2.3. Proximate Composition Analysis

Analytical determinations for proximate composition analysis of dried *S. horneri* samples were analyzed based on the method proposed by the Association of Official Analytical Chemists [27]. The samples were dried at 110 °C to determine the moisture content (AOAC method 940.05). Crude protein content was analyzed using the Kjeldahl procedure (AOAC method 954.01). Crude fat content was analyzed by extracting an ether-soluble substance (AOAC method 920.39). Crude ash content was analyzed by sample incineration in a muffle furnace at 600 °C (AOAC method 942.05). Carbohydrate content was analyzed as follows:

Total carbohydrate (%) = 100 − (% moisture + % protein +% lipid + % ash).

### 2.4. Monosaccharide Composition Analysis

To determine the monosaccharide composition, the polysaccharide was hydrolyzed using 4M trifluoroacetic acid. Next, it was separated using a CarboPac PA1 column integrated with a Dionex ED50 detector (HPAEC-PAD; Dionex, Sunnyvale, CA, USA). Standardized monosaccharide mixtures were prepared using fucose, rhamnose, arabinose, glucose, fructose, galactose, and xylose.

### 2.5. High-Performance Liquid Chromatography (HPLC) Analysis of S. horneri

The *S. horneri* 70% ethanol extract was precisely quantified to 9.86 mg, dissolved in 1 mL of methanol, and then filtered to obtain an extract sample. The HPLC and HPLC column (YMC-Triart C18, 4.6 × 250 mm, 5 μm, ThermoFisher, Waltham, MA, USA), consisting of a quaternary HPLC pump (LPG-3400SD, ThermoFisher) and a diode array detector (DAD-3000, ThermoFisher), were connected and used for analysis. Mobile solvent systems (isocratic, A channel:B channel = 3:97), 0.1% acetic acid distilled water (A channel), and methanol (B channel) were used. During the analysis, the flow rate was set to be maintained at 1 mL/min. Next, 50 μL of the 9.86 mg/mL *S. horneri* 70% ethanol extract solution was injected. Then, after dissolving 50 μL of 2.5 mg/mL fucosterol (≥98%, AktinChemicals Inc., Chengdu, China) in methanol, it was used for analysis as a standard sample. The detection wavelength was set to 210 nm. A calibration graph was created by setting different concentrations of fucosterol (10, 25, 50, 100, and 500 μg/mL), and then quantitative analysis was performed using a calibration graph.

### 2.6. DPPH Radical Scavenging Activity

First, DPPH solution (0.20 mM) was dissolved in methanol. Samples of various concentrations (0.0625, 0.125, 0.25, 0.5, 1, 2, and 4 mg/mL) were prepared. Then, 150 μL of DPPH solution was mixed with 50 μL of each sample. After 15 min of incubation in a dark room, the decrease in the absorbance of the solution was measured at 517 nm. Inhibitory activity against DPPH was expressed by the percentage inhibition (%) in the above assay system using the following formula, where A_sample_ is the absorbance of the test sample and A_control_ is the absorbance of the control.

DPPH radical scavenging activity (%) = (A_control_ − A_sample_)/A_control_ × 100

### 2.7. ABTS Radical Scavenging Activity

The ABTS reagent was prepared by mixing ABTS (7 mM) and potassium persulfate (140 mM). To induce free radical formation, the mixture was kept in the dark for 16 h at room temperature and diluted with water. Then, 100 μL of sample was mixed with 100 μL ABTS reagent in a 96-well microplate. After incubation at room temperature for 6 min, the absorbance was measured at 734 nm, and 100% methanol was used as a control. ABTS scavenging activity was measured using the following formula:

ABTS radical scavenging activity (%) = (Blank O.D. − Sample O.D.)/Blank O.D. × 100

### 2.8. Cell Culture and Viability Assay

BV2 cells (5 × 10^5^ cells/mL) were cultured in RPMI-1640 containing 10% heat-inactivated fetal bovine serum and Antibiotic-Antimycotic (ThermoFisher, Waltham, MA, USA). HT22 cells were maintained in DMEM supplemented with same components as BV2 cells. Cells were incubated at 37 °C in 5% CO_2_. The effect of *S. horneri* extract and its fractions on cell viability was evaluated by measuring mitochondrial reductase function. This is based on the principle that tetrazolium salt 3-[4,5-dimethylthiazol-2-yl]-2,5-diphenyltetrazolium bromide (MTT) is reduced to formazan crystals. For the determination of cell viability, 5 mg/mL of MTT was mixed with each cell suspension (1 × 10^5^ cell/mL per well of the 48-well plates) for 4 h. The formazan crystals in the cells were dissolved in DMSO, and then the optical density of each sample group solution was confirmed at a wavelength of 540 nm.

### 2.9. Determination of Nitrite Levels

Nitrite was measured as an indicator of NO production in cells. Briefly, a method based on the reaction of Griess reagent (Sigma-Aldrich Chemical Co., St. Louis, MO, USA) was used to determine the nitrite production using the conditioned medium [28]. The details of determination of nitrite levels have been described previously [29].

### 2.10. Determination of PGE_2_ Levels

The level of PGE_2_ was measured using the specific ELISA kit from R&D Systems, Inc. (Minneapolis, MN, USA), according to a previously described method [29]. Briefly, BV2 cells were cultured in 48-well plates (1 × 10^5^ cell/mL) and pre-incubated with different concentrations of *S. horneri* extract and its fractions for 3 h. Subsequently, BV2 cells were induced by LPS (1 μg/mL for 24 h). To remove particulate matter, the supernatants were collected and then centrifuged at 13,000× *g* for 2 min. After that, the levels of PGE_2_ were measured using the ELISA kit according to the protocol in the instructions provided by the manufacturer.

### 2.11. Determination of IL-6 and TNF-α Levels

The levels of IL-6 and TNF-α were measured using specific ELISA kits (R&D Systems, Inc.), according to the protocol in the instructions provided by the manufacturer. Briefly, BV2 cells (5 × 10^5^ cells/well) were seeded in 48-well culture plates and pre-incubated with different concentrations of *S. horneri* extract and its fractions for 3 h. Subsequently, cells were stimulated with LPS (1 μg/mL) for 24 h. After incubation, the levels of IL-6 and TNF-α were measured using the collected supernatant of medium by cytokine ELISA kits according to the protocol in the instructions provided by the manufacturer.

### 2.12. Western Blot Analysis

NF-κB p65, iNOS, COX-2, HO-1, and Nrf2 protein levels were determined by Western blot analysis. To perform whole-cell lysis, cells were lysed using a protease inhibitor mixture (0.1 mM PMSF, 1 mg/mL chymostatin, 5 mg/mL aprotinin, and 5 mg/mL pepstatin A) and 20 mM Tris-HCl buffer (pH 7.4). Protein Assay Dye Reagent Concentrate (#5000006; Bio-Rad Laboratories, Hercules, CA, USA) was used to measure the protein concentration. Western blotting was performed as previously described [29].

### 2.13. Preparation of Cytosolic and Nuclear Fractions

Nuclear Extraction Kit (Cayman, Ann Arbor, MI, USA) was used to obtain the cytosolic and nuclear fractions. Each extracted fraction was lysed according to the protocol in the instructions provided by the manufacturer.

### 2.14. DNA-Binding Activity of NF-κB

NF-κB DNA binding activity was measured using nuclear extracts from cells. Then, it was analyzed with the NF-κB Transcription Factor Assay kit (Cayman, Ann Arbor, MI, USA). Detailed analysis methods were performed according to the protocol in the instructions provided by the manufacturer.

### 2.15. NF-κB Localization and Immunofluorescence

BV2 cells were cultured using Lab-Tek II chamber slides and then treated with *S. horneri* extract and its fractions (100 μg/mL) for 3 h before stimulation by LPS (1 μg/mL) for 1 h. Cells were fixed with formalin and then permeated with cold acetone. Next, cells were irradiated with p65 antibody and then incubated with fluorescein isothiocyanate (FITC)-labeled secondary antibody (Alexa Fluor 488, Invitrogen, Carlsbad, CA, USA). For nuclear staining, cells were treated with 1 μg/mL of 4′,6-diamidino-2-phenylindole (DAPI) for 30 min. Vectashield (Vector Laboratories, Burlingame, CA, USA) was treated after washing with PBS. Pictures of stained cells were confirmed using a Zeiss fluorescence microscope (Provis AX70; Olympus Optical Co., Tokyo, Japan) [30].

### 2.16. Reactive Oxygen Species Generation Assays

HT22 cells were treated with glutamate (10 mM for 12 h) and/or CH_2_Cl_2_-soluble fraction (12.5–50 μg/mL). After 12h, HT22 cells were washed with PBS and incubated in Hank’s balanced salt solution with 10 μM of 2′,7′-dichlorofluorescein diacetate (DCFDA). The cells were then stored in darkness for 1 h. Then, the medium was removed, and the cells were extracted using 1% Triton X-100 in PBS. Fluorescence at 490 nm and 525 nm was measured using a SpectraMax Gemini XS (Molecular Devices, Sunnyvale, CA, USA).

### 2.17. Statistical Analysis

All results in the present study were obtained via at least 3 independent experiments. The data were expressed as the mean ± standard deviation of 3 independent experiments. All data were evaluated using one-way analysis of variance (ANOVA) followed by Tukey’s multiple comparison test and compared to 3 or more groups. Statistical analyses were performed using GraphPad Prism (Version 5.01, GraphPad Software, Inc., San Diego, CA, USA).

## 3. Results

### 3.1. Proximate Composition of S. horneri and Composition of Monosaccharides from S. horneri Extract and Its Fractions

The proximate composition, including moisture, carbohydrate, ash, crude fat, and crude protein content of *S. horneri* was analyzed. *S. horneri* contained high levels of carbohydrate (45.99 ± 1.61%), ash (20.93 ± 0.41%), crude protein (14.52 ± 1.06%), moisture (14.19 ± 0.39%), and crude fat (4.37 ± 0.14%) (Table 1).

**Figure 1:**
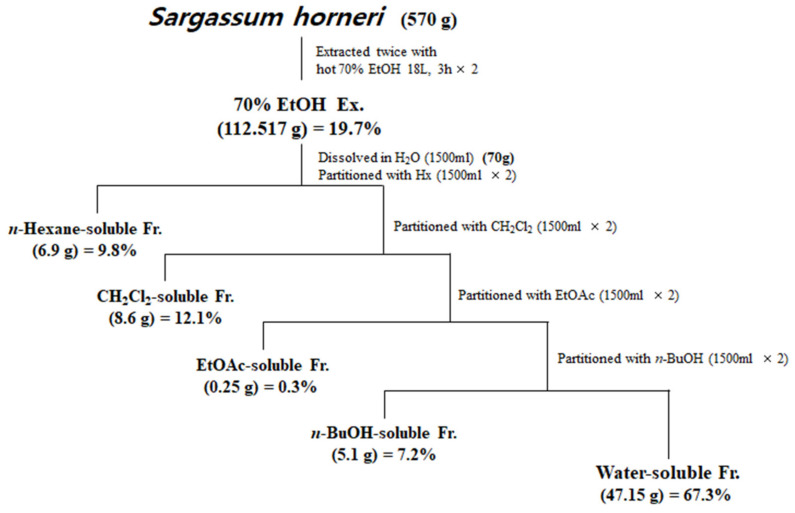
Preparation of *S. horneri* extract and its partitioned fractions.

Next, the dried *S. horneri* was extracted with 70% EtOH and then partitioned sequentially with equal volumes of *n-*hexane, dichloromethane (CH_2_Cl_2_), ethyl acetate (EtOAc), and *n*-butanol (*n*-BuOH) (Figure 1). The yields of sub-fractions were low in the hexane- (9.8%), EtOAc- (0.3%), and BuOH-soluble fractions (7.2%) compared to those of the CH_2_Cl_2_- (12.1%) and water-soluble fractions (67.3%). Therefore, we analyzed the monosaccharide composition of the 70% EtOH extract of *S. horneri* and its CH_2_Cl_2_- and water-soluble fractions. According to the monosaccharide composition analysis, 70% EtOH extract contained high levels of galactose (56.75%), followed by glucose (13.69%), fucose (12.94%), arabinose (2.80%), and rhamnose (1.27%). However, xylose and fructose were not detected (Table 2). Moreover, the CH_2_Cl_2_-soluble fraction contained high levels of galactose (82.82%), followed by fucose (9.35%), arabinose (0.54%), and rhamnose (0.61%), but glucose, xylose, and fructose were not detected (Table 2). In addition, the water-soluble fraction contained high levels of glucose (32.99%), followed by fucose (21.61%), galactose (20.40%), arabinose (3.70%), and rhamnose (2.98%), but xylose and fructose were not detected (Table 2).

### 3.2. HPLC Analysis of 70% EtOH Extract of S. horneri

**Figure 2:**
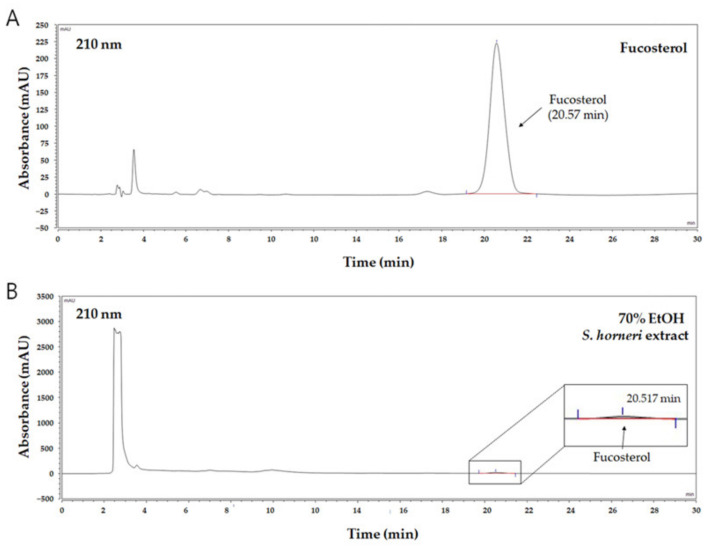
HPLC chromatogram of fucosterol as a standard compound and 70% EtOH *S. horneri* extract. (**A**) Fucosterol, (**B**) 70% EtOH *S. horneri* extract.

It is well-known that brown algae including *S. horneri* contain abundant sterol type compounds such as fucosterol [31]. Fucosterol was detected in the HPLC chromatogram (UV wavelength: 210 nm) at around 20.6 min of retention time (Figure 2A), and concentration ranges of 10–500 µg/mL were prepared with the calibration curve. Based on the HPLC analysis of the 70% EtOH extract of *S. horneri*, the area of the peak corresponding to fucosterol in the chromatogram was substituted for the fucosterol calibration curve to analyze the contents. Consequently, it was found that this extract contained approximately 34.37 ± 0.75 μg/mg fucosterol (Figure 2B).

### 3.3. Effects of S. horneri Extract and Its Fractions on DPPH and ABTS Radical Scavenging Activity

**Figure 3:**
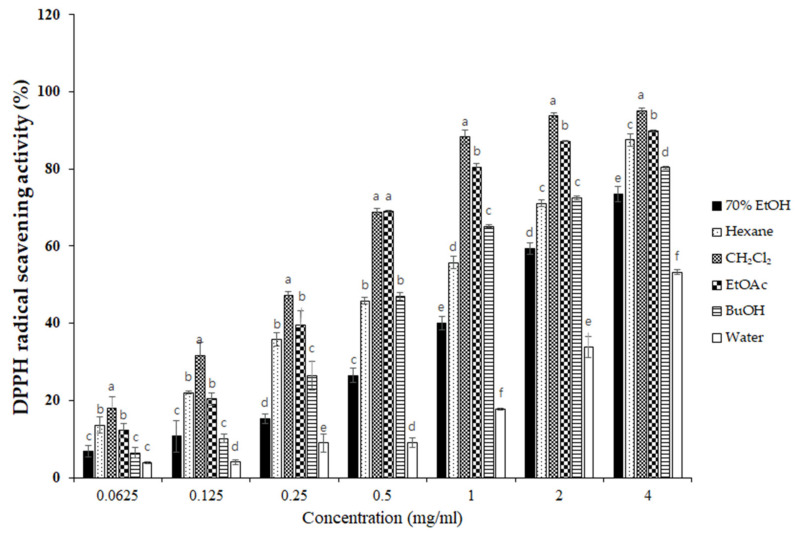
Effects of *S. horneri* extract and its fractions on DPPH radical scavenging activity. The measurement of DPPH radical scavenging activity is described in the Materials and Methods section. All data represent mean values of 3 independent experiments ±SD. Data were statistically considered at *p* < 0.05, and different letters (a–f) in graph represent statistical difference.

**Figure 4:**
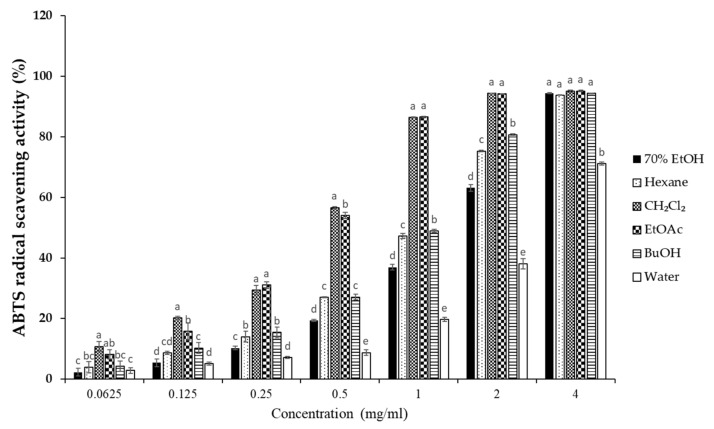
Effects of *S. horneri* extract and its fractions on ABTS radical scavenging activity. The measurement of ABTS radical scavenging activity is described in the Materials and Methods section. All data represent mean values of 3 independent experiments ±SD. Data were statistically considered at *p* < 0.05, and different letters (a–e) in graph represent statistical difference.

To elucidate the antioxidant activity of *S. horneri* extract and its fractions, we performed radical scavenging assays using 2,2-diphenyl-1-picrylhydrazyl (DPPH) and 2,2′-azino-bis(3-ethylbenzothiazoline-6-sulfonic acid (ABTS). DPPH and ABTS free radical scavenging assays are often used to evaluate the antioxidant potential of compounds or extracts. These are rapid, simple, and widely used methods for testing antioxidant activity. The DPPH and ABTS radical scavenging activities of *S. horneri* extract and its fractions were increased (Figure 3 and Figure 4). Among them, the CH_2_Cl_2_- and EtOAc-soluble fractions showed superior potency in terms of DPPH and ABTS radical scavenging activities, and the CH_2_Cl_2_-soluble fraction showed the most remarkable effects (Figure 3 and Figure 4).

### 3.4. Effects of S. horneri Extract and Its Fractions on the Viability of BV2 Cells

**Figure 5:**
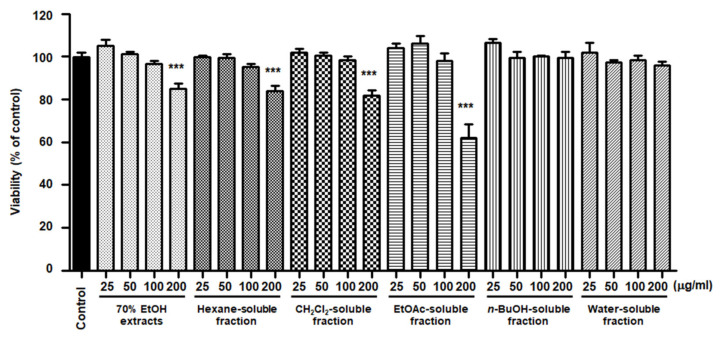
Effects of 70% EtOH extract and its *n*-Hexane-, CH_2_Cl_2_-, EtOAc-, *n*-BuOH-, and water-soluble fractions on BV2 cell viability. The cells were treated with *S. horneri* extract and its fractions for 48 h. Bars represent the mean ± standard deviation of 3 independent experiments. *** *p* < 0.001. Control means an untreated control group.

To elucidate the effects of *S. horneri* extract and its fractions on BV2 cells, cells were initially seeded in microplates, followed by treatment with *S. horneri* extracts and fractions (25–200 μg/mL). At concentrations of up to 200 μg/mL, BuOH- and water-soluble fractions had no effect on cell viability, but the 70% EtOH extract (85 ± 4.3%), hexane (84 ± 4.0%)-, CH_2_Cl_2_ (81.6 ± 4.72%)-, and EtOAc (62.5 ± 10.8%)-soluble fractions had toxic effects at 200 μg/mL (Figure 5). Thus, a non-toxic concentration range was selected for subsequent studies on the anti-inflammatory effects (70% EtOH extract, hexane-, CH_2_Cl_2_-, EtOAc-soluble fractions at 100 μg/mL, and *n*-BuOH-, water-soluble fractions at 200 μg/mL).

### 3.5. Effects of S. horneri Extract and Its Fractions on the Production of Pro-Inflammatory Mediators and Cytokines in LPS-Induced BV2 Cells

**Figure 6:**
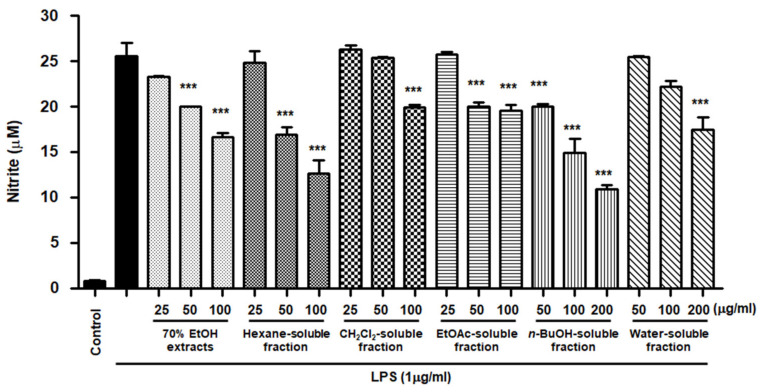
Effects of 70% EtOH extract and its *n*-Hexane-, CH_2_Cl_2_-, EtOAc-, *n*-BuOH-, and water-soluble fractions on nitrite content in BV2 cells. Cells were pretreated for 3 h with indicated concentrations of *S. horneri* extract and its fractions, and stimulated for 24 h with LPS (1 μg/mL). The measurement of nitrite concentrations is described in the Materials and Methods section. Bars represent the mean ± standard deviation of 3 independent experiments. *** *p* < 0.001 compared with LPS-treated group. Control means an untreated control group.

NO is a pro-inflammatory mediator that is increasingly expressed under inflammatory pathological conditions by activated microglia. The cells were pretreated with *S. horneri* extract and its fractions at concentrations of 25–200 μg/mL and then induced by LPS. As demonstrated in Figure 6, cells treated with *S. horneri* extract and its fractions showed decreased NO production compared to cells treated with LPS alone (25.53 ± 2.5 μM); 70% EtOH extract (16.67 ± 0.77 μM at 100 μg/mL), hexane (12.64 ± 2.5 μM at 100 μg/mL)-, CH_2_Cl_2_ (19.89 ± 0.4 μM at 100 μg/mL)-, EtOAc (19.59 ± 1.0 μM at 100 μg/mL)-, *n*-BuOH (10.93 ± 0.7 μM at 200 μg/mL)-, and water (17.46 ± 2.3 μM at 200 μg/mL)-soluble fractions.

**Figure 7:**
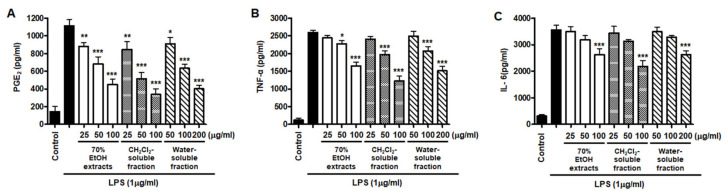
Effects of 70% EtOH extract and its CH_2_Cl_2_- and water-soluble fractions on the levels of PGE_2_ (**A**), TNF-α (**B**), and IL-6 (**C**) in BV2 cells. Cells were pretreated for 3 h with the indicated concentrations of *S. horneri* extract and its fractions, and stimulated for 24 h with LPS (1 μg/mL). The methods used to measure the PGE_2_, TNF-α, and IL-6 levels are described in detail in the Materials and Methods section. Bars represent the mean ± standard deviation of 3 independent experiments. * *p* < 0.05, ** *p* < 0.01, *** *p* < 0.001 compared with LPS-treated group. Control means an untreated control group.

However, low yields were observed in the hexane- (9.8%), EtOAc- (0.3%), and BuOH-soluble fractions (7.2%) compared to those of CH_2_Cl_2_- (12.1%) and water-soluble fractions (67.3%) (Figure 1). Therefore, subsequent experiments were only performed using *S. horneri* extract and the CH_2_Cl_2_- and water-soluble fractions. Next, the inflammatory mediators and cytokines were analyzed. PGE_2_ is a key molecule involved in inflammation or neuroinflammation. When BV2 cells were induced by LPS, PGE_2_ levels increased significantly in LPS treatment (1112.9 ± 70.07 pg/mL); however, treatment with 70% EtOH extract (451.0 ± 61.8 pg/mL at 100 μg/mL) and CH_2_Cl_2_ (337.1 ± 63.92 pg/mL at 100 μg/mL)- and water (402.8 ± 40.92 pg/mL at 200 μg/mL)-soluble fractions inhibited PGE_2_ levels (Figure 7A). Next, BV2 cells were induced by LPS in the absence or presence of *S. horneri* extract or CH_2_Cl_2_- or water-soluble fractions, and the levels of TNF-α and IL-6 were determined. The results showed that treatment with *S. horneri* extract and its fractions decreased the LPS-induced TNF-α production (LPS treatment: 2592.5 ± 64.5 pg/mL, 70% EtOH extract at 100 μg/mL: 1654.8 ± 98.2 pg/mL, CH_2_Cl_2_-soluble fractions at 100 μg/mL: 1226.2 ± 145.2 pg/mL, and water-soluble fractions at 200 μg/mL: 1514.7 ± 126.4 pg/mL) and IL-6 production (LPS treatment: 3555.5 ± 175.9 pg/mL, 70% EtOH extract at 100 μg/mL: 2621.8 ± 227.5 pg/mL, CH_2_Cl_2_-soluble fractions at 100 μg/mL: 2176.8 ± 235.7 pg/mL, and water-soluble fractions at 200 μg/mL: 2627.4 ± 138.9 pg/mL) (Figure 7B,C).

**Figure 8:**
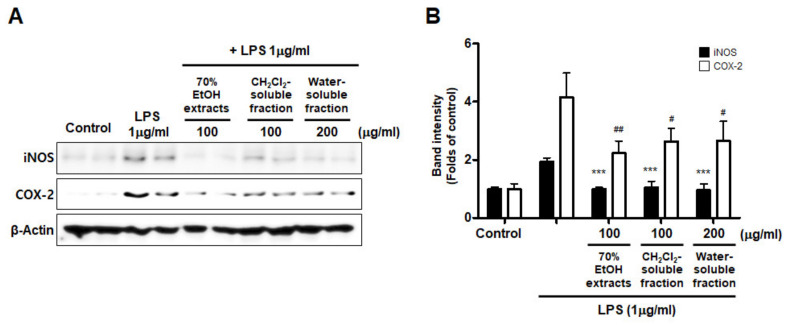
Effects of 70% EtOH extract and its CH_2_Cl_2_- and water-soluble fractions on iNOS and COX-2 protein levels in BV2 cells. (**A**) Cells were treated with the indicated concentrations of 70% EtOH extract and its CH_2_Cl_2_- and water-soluble fractions for 3 h, and then stimulated for 24 h with LPS (1 μg/mL). The method used to perform the Western blot analysis is described in detail in the Materials and Methods section. (**B**) Immunoblots were quantified using ImageJ software. Band intensity was normalized to β-actin. *** *p* < 0.001, # *p* < 0.05, ## *p* < 0.01, compared to LPS-treated group. Control means an untreated control group.

Furthermore, we investigated whether this inhibitory effect on inflammatory mediators and cytokines was involved in iNOS and COX-2 regulation. The results appeared to show that LPS treatment increased iNOS and COX-2 induction, but pre-treatment with *S. horneri* extract and its fractions suppressed the LPS-induced increase in iNOS and COX-2 levels (Figure 8).

### 3.6. Effects of S. horneri Extract and Its CH_2_Cl_2_- and Water-Soluble Fractions on NF-κB Activation in LPS-Induced BV2 Cells

**Figure 9:**
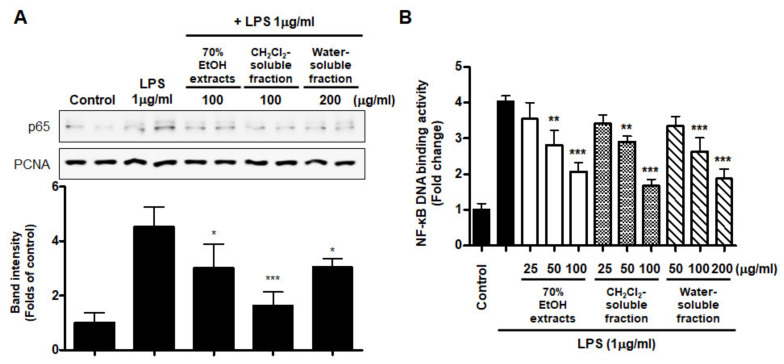
Effects of 70% EtOH extract and its CH_2_Cl_2_- and water-soluble fractions on NF-κB p65 activation (**A**) and NF-κB DNA-binding activity (**B**) in BV2 cells. Cells were pretreated with the indicated concentrations of 70% EtOH extract and its CH_2_Cl_2_- and water-soluble fractions for 3 h and stimulated with LPS (1 μg/mL) for 1 h. The method used to perform the Western blot analysis for nuclear NF-κB translocation is described in detail in the Materials and Methods section. Immunoblots were quantified using ImageJ software. Band intensity was normalized to PCNA. A commercially available NF-κB ELISA kit was used to test the nuclear extracts and determine the NF-κB binding levels. Data are the mean values of 3 independent experiments. * *p* < 0.05, ** *p* < 0.01, *** *p* < 0.001 compared to LPS-treated group. Control means an untreated control group.

Abnormal NF-κB signaling during the inflammatory response leads to excessive production of pro-inflammatory mediators and cytokines. To evaluate whether NF-κB inactivation plays a role in mediating the effect of *S. horneri* extract and its fractions, we assessed the inhibitory effect of *S. horneri* extract and its fractions on LPS-stimulated NF-κB activation. We used nuclear fractions obtained from BV2 cells pretreated with or without *S. horneri* extract and fractions (50–100 μg/mL) and then induced with LPS. In the results of Figure 9A, pretreatment with 70% EtOH extract (50 and 100 μg/mL) and CH_2_Cl_2_- and water-soluble fractions (50 and 100 μg/mL) inhibited the nuclear translocation of p65. In addition, the increase in DNA binding activity of NF-κB by LPS was attenuated by 70% EtOH extract and the CH_2_Cl_2_- and water-soluble fractions (LPS treatment: 4.03 ± 0.15 Fold, 70% EtOH extract at 100 μg/mL: 2.07 ± 0.24 Fold, CH_2_Cl_2_-soluble fraction at 100 μg/mL: 1.67 ± 0.19 Fold, and water-soluble fraction at 200 μg/mL: 1.87 ± 0.26 Fold) (Figure 9B).

**Figure 10:**
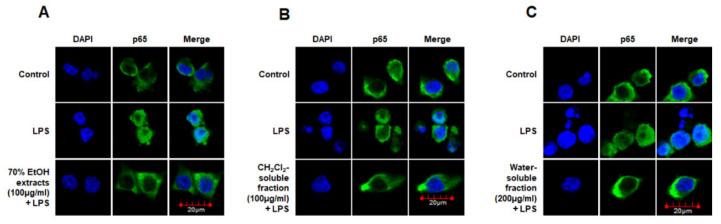
Effects of 70% EtOH extract (**A**) and its CH_2_Cl_2_- (**B**) and water-soluble fractions (**C**) on NF-κB localization in BV2 cells. Cells were pretreated for 3 h with the indicated concentrations of this extract and its fractions, and then stimulated for 1 h with LPS (1 μg/mL). The method used to perform the immunofluorescent analysis is described in detail in the Materials and Methods section. Control means an untreated control group.

Moreover, we analyzed the nuclear translocation of p65 in cells treated with *S. horneri* extract and fractions, LPS, or both using an anti-p65 FITC-labeled antibody. DAPI was used for nuclear staining. In the control group, p65 expression was detected in the cytosol. However, in the LPS-induced cells, p65 accumulation was detected in the nucleus, as indicated in the merged images of DAPI and p65 staining. Furthermore, in LPS-stimulated cells pretreated with *S. horneri* extract and its fractions, p65 distribution was detected throughout the cytosol, similar to the control group (Figure 10). These findings indicate that *S. horneri* extract and its fractions are negative regulators of LPS-stimulated NF-κB nuclear translocation.

### 3.7. Effects of S. horneri Extract and Its CH_2_Cl_2_- and Water-Soluble Fractions on HO-1 Expression and Nrf2 Nuclear Translocation

**Figure 11:**
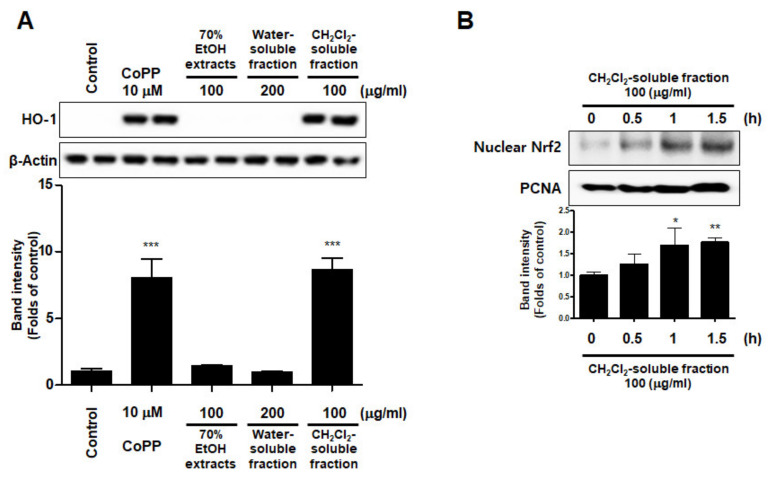
Effects of 70% EtOH extract and its water- and CH_2_Cl_2_-soluble fractions on the protein expression levels of HO-1 (**A**) and the nuclear translocation of nuclear factor erythroid-derived 2-related factor-2 (Nrf2) by the CH_2_Cl_2_-soluble fraction (**B**) in BV2 cells. (**A**) Cells were incubated for 12 h with the indicated concentration of each sample. (**B**) These cells were treated with CH_2_Cl_2_-soluble fraction for 0.5, 1, or 1.5 h, and then the nuclei were fractionated from the cytosol using a Cayman Nuclear Extraction Kit. HO-1 and nuclear Nrf2 expression were then determined by Western blotting. Band intensities were normalized to β-actin or PCNA. Cobalt protoporphyrin (CoPP, 10 μM) was used as a positive control. Data are presented as the mean ± standard deviation of 4 (**A**) or 3 (**B**) independent experiments. * *p* < 0.05, ** *p* < 0.01, *** *p* < 0.001 compared to the control group. Control means an untreated control group.

**Figure 12:**
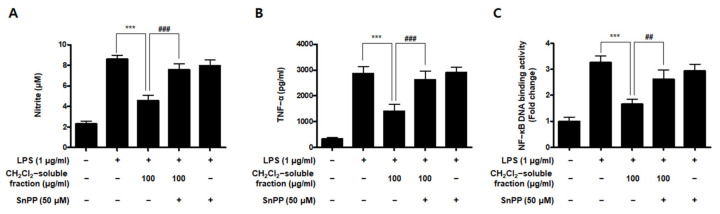
Effects of CH_2_Cl_2_-soluble fraction-induced Nrf2-mediated HO-1 expression on LPS-stimulated proinflammatory mediator production in BV2 cells. Cells were pretreated with the CH_2_Cl_2_-soluble fraction for 3 h with or without SnPP (50 μM), and subsequently stimulated with LPS (1 μg/mL) for 24 h. In the Materials and Methods section, the methods used for measurement of nitrite (**A**), TNF-α (**B**), and NF-κB DNA-binding activity (**C**) are described in detail. Data are presented as the mean ± standard deviation for 3 independent experiments. *** *p* < 0.001, ## *p* < 0.01, and ### *p* < 0.001.

To investigate whether *S. horneri* extract and its CH_2_Cl_2_- and water-soluble fractions induced the HO-1 expression, cells were induced with *S. horneri* extract and its CH_2_Cl_2_- and water-soluble fractions for 12 h. Cells pre-treated with cobalt protoporphyrin (CoPP) were used as an HO-1 inducer. The CH_2_Cl_2_-soluble fraction significantly induced HO-1 expression in BV2 cells (Figure 11A). Thus, we examined the effect of the CH_2_Cl_2_-soluble fraction on the nuclear translocation of activated Nrf2. BV2 cells were treated with 100 μg/mL CH_2_Cl_2_-soluble fraction, and Nrf2 expression was determined. The CH_2_Cl_2_-soluble fraction treatment induced nuclear translocation of Nrf2 (Figure 11B). To confirm whether the anti-neuroinflammatory effects of the CH_2_Cl_2_-soluble fraction were related to HO-1 regulation, we conducted further experiments with tin protoporphyrin-IX (SnPP), a selective HO-1 inhibitor. After pretreatment with the CH_2_Cl_2_-soluble fraction with or without 50 μM SnPP, the cells were treated with LPS for 24 h. Pretreatment with the CH_2_Cl_2_-soluble fraction resulted in decreased nitrite production (LPS treatment: 8.5 ± 0.3 μM, CH_2_Cl_2_-soluble fractions: 4.5 ± 0.4 μM, and SnPP with CH_2_Cl_2_-soluble fractions: 7.59 ± 0.5 μM), TNF-α production (LPS treatment: 2862.2 ± 271.9 μg/mL, CH_2_Cl_2_-soluble fractions: 1402.7 ± 262.0 μg/mL, and SnPP with CH_2_Cl_2_-soluble fractions: 2629.1 ± 328.3 μg/mL), and NF-κB DNA-binding activity (LPS treatment: 3.72 ± 0.24 Fold, CH_2_Cl_2_-soluble fractions: 1.66 ± 0.18 Fold, and SnPP with CH_2_Cl_2_-soluble fractions: 2.61 ± 0.35 Fold) in LPS-induced BV2 cells (Figure 12). However, the anti-inflammatory action of the CH_2_Cl_2_-soluble fraction was changed by SnPP. SnPP did not affect nitrite, TNF-α, or NF-κB DNA-binding activity following LPS stimulation. These findings indicate that the anti-neuroinflammatory action of the CH_2_Cl_2_-soluble fraction is regulated by HO-1-related signaling.

### 3.8. Effect of CH_2_Cl_2_-Soluble Fraction Treatment on Glutamate-Induced Oxidative Damage and Reactive Oxygen Species (ROS) Production in HT22 Cells

**Figure 13:**
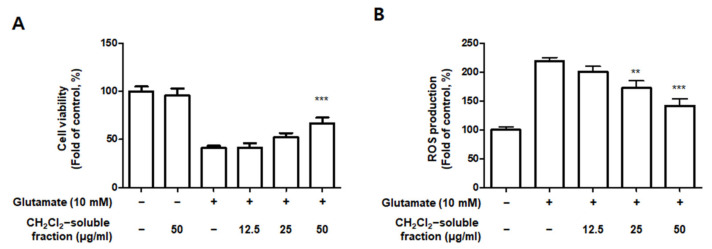

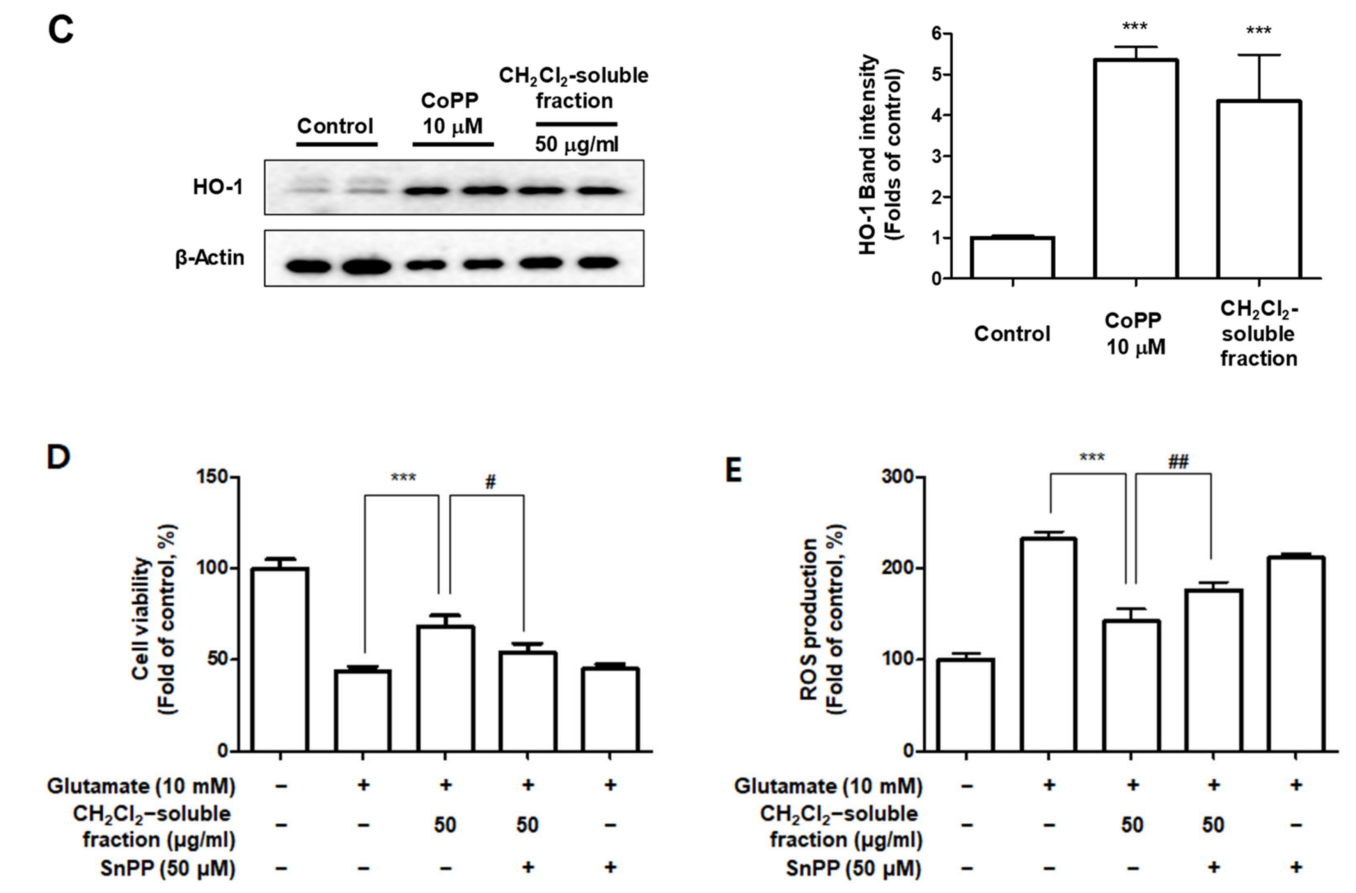
Effect of CH_2_Cl_2_-soluble fraction treatment on cell viability (**A**), ROS generation (**B**), and HO-1 expression (**C**) in HT22 cells, and effects of HO-1 induction by CH_2_Cl_2_-soluble fraction on glutamate-induced oxidative neurotoxicity (**D**) and ROS generation (**E**). (**A**,**B**) HT22 cells were treated with CH_2_Cl_2_-soluble fractions (12.5, 25, and 50 μM) for 3 h and then incubated with glutamate (10 mM) for 12 h. ** *p* < 0.01, *** *p* < 0.001 compared to glutamate-treated cells. (**C**) Cells were incubated for 12 h with CH_2_Cl_2_-soluble fractions (50 μg/mL) and analyzed using Western blotting. Band intensities were normalized to β-actin. CoPP (10 μM) was used as a positive control. *** *p* < 0.001 when compared to the control group. (**D**,**E**) HT22 cells were treated with CH_2_Cl_2_-soluble fraction (50 μg/mL) in the presence or absence of SnPP (50 μM). (**D**) Cell viability and (**E**) ROS generation were measured following treatment with glutamate (10 mM) for 12 h. *** *p* < 0.001, # *p* < 0.05, and ## *p* < 0.01. All data are presented as the mean ± standard deviation for 3 independent experiments.

Based on the increased HO-1 levels induced by CH_2_Cl_2_-soluble fractions in BV2 cells, we determined the protective properties or anti-oxidative stress caused by CH_2_Cl_2_-soluble fraction pre-treatment followed by glutamate stimulation in HT22 cells. HT22 cells were treated with CH_2_Cl_2_-soluble fractions and challenged to glutamate. Pretreatment of HT22 cells with 12.5–50 μg/mL of CH_2_Cl_2_-soluble fraction significantly attenuated the effects of glutamate-induced oxidative cell death (glutamate: 40.95 ± 2.33%, CH_2_Cl_2_-soluble fraction at 50 μg/mL: 66.97 ± 5.77%) (Figure 13A). In addition, pretreatment with 12.5–50 μg/mL CH_2_Cl_2_-soluble fraction also significantly reduced ROS production induced by glutamate (glutamate: 218.9 ± 6.7%, CH_2_Cl_2_-soluble fraction at 50 μg/mL: 141.7 ± 12.6%) (Figure 13B). Next, we investigated whether HO-1 levels were induced in HT22 cells incubated with CH_2_Cl_2_-soluble fraction. As a result, we confirmed that this fraction markedly induced HO-1 expression (Figure 13C). To determine whether the neuroprotective capacity of CH_2_Cl_2_-soluble fractions was correlated with HO-1 expression in HT22 cells, we included a set of experiments with SnPP. After the cells were treated with CH_2_Cl_2_-soluble fractions with or without SnPP, the cells were treated with glutamate for 12 h. The pretreatment with CH_2_Cl_2_-soluble fractions resulted in protecting HT22 cells (Figure 13D) and reducing ROS production (Figure 13E) in glutamate-induced HT22 cells. However, the neuroprotective effects and ROS reduction attributable to the CH_2_Cl_2_-soluble fraction were also reversed by SnPP (Figure 13D,E). This result suggests that the neuroprotective effects of CH_2_Cl_2_-soluble fractions are regulated by HO-1 levels.

## 4. Discussion

In the marine environment, seaweed creates a suitable habitat for marine life including fish, turtles, sea birds, and invertebrates. It also provides spawning grounds for these animals and nursery areas for many other marine organisms [32,33]. Seaweed algae are classified into four groups: green algae (Chlorophyceae), red algae (Rhodophycae), blue-green algae (Cyanophyceae), and brown algae (Phaeophyceae). *Sargassum* is a genus of brown algae that contains approximately 400 species belonging to the Sargassaceae family [12]. *S. horneri* is an annual brown alga found throughout the waters off the coast of China, Japan, and Korea. However, it was recently reported that large biomasses of *S. horneri* drifted and accumulated along the southern coast of Korea and Jeju Island in 2015 [16]. Therefore, a treatment plan is required for the excess accumulation of seaweed biomass. Accordingly, it is necessary to investigate the biological activities of these edible seaweeds and possibly identify therapeutic agents. In the present study, we performed a biological evaluation of *S. horneri* extract and its fractions, and assessed their antioxidant and anti-neuroinflammatory effects on LPS-induced BV2 cells.

First, we determined the chemical composition of S. *horneri*. In addition, 70%EtOH extract of *S. horneri* contained approximately 34.37 ± 0.75 μg/mg fucosterol. From the results of this study, fucosterol could be identified as an indicator component in the 70% EtOH extract of *S. horneri*. This result is important to development of products using the 70% EtOH extract of *S. horneri*. Fucosterol can be used as an important index component for quality control in the development of products using the 70% EtOH extract of *S. horneri*. We analyzed the proximate composition of *S. horneri* raw material. The raw material of *S. horneri* consisted mostly of carbohydrate (45.99 ± 1.61%). A previous study reported that *S. horneri* collected from Jeju, the same region from which we collected our samples in this study, consisted mostly of polysaccharide (60.43 ± 3.31) [34]. This difference is thought to be due to different collection seasons. Another previous study also reported that the proximate composition varied depending on the collection season [35]. Subsequently, the monosaccharide composition of *S. horneri* extracts and fractions that had excellent anti-neuroinflammatory effects was studied. According to the monosaccharide composition analysis, the 70% EtOH extract contained high levels of galactose (56.75%) as did the CH_2_Cl_2_-soluble fraction (82.82%), and the water-soluble fraction contained high levels of glucose (32.99%). A previous study reported the monosaccharide composition of *S. horneri* using only water extracts [36]. This is the first report on the monosaccharide composition of *S. horneri* using 70% EtOH extract and its fractions. Therefore, we suggest that the extraction and fractionation method used in our study is meaningful in that it presents a novel approach.

Next, we performed DPPH and ABTS assays to determine the antioxidant capacities of the *S. horneri* extract and its fractions. DPPH and ABTS assays are among the most common antioxidant capacity assays. The DPPH radical scavenging assay is a method of measuring reducing power using the electron-donating ability of DPPH, a relatively stable radical. DPPH can be a stable or diamagnetic molecule by receiving electrons or hydrogen radicals. Compounds that can react with DPPH radicals are generally strong hydrogen donors. In addition, compounds with multiple hydroxyl groups might be able to serve a role as hydrogen donors [37]. ABTS is frequently used as a substrate with hydrogen peroxide as the enzyme of peroxidase. Its use makes it possible to follow the reaction kinetics of peroxidases. Therefore, the ABTS analysis indirectly follows the kinetics of the hydrogen peroxide-producing enzyme. It is also commonly used in experiments that simply quantify the amount of hydrogen peroxide in natural product extracts or natural compounds [38]. According to the special chemical properties of the formed free radicals, DPPH and ABTS assays are used to measure the antioxidant capacity of natural products. In this study, the CH_2_Cl_2_-soluble fraction showed the most remarkable action on the DPPH and ABTS free radical scavenging activity among the *S. horneri* extract and all fractions (Figure 2 and Figure 3).

NO is a known regulator and mediator of inflammatory responses. This small molecule is synthesized from l-arginine and catalyzed by isoforms of the NOS enzyme [39]. There are three isoforms of NOS: iNOS, neuronal NOS, and endothelial NOS. PGE_2_ is a biologically active small molecule that plays an important role in the regulation of inflammation [40]. PGE_2_ is a derivative of arachidonic acid produced by the constitutive COX-1 or inducible COX-2 enzymes and consists of constitutive COX-1 and inducible COX-2 [41]. NO and PGE_2_ are important inflammatory mediators that are involved in various regulatory functions during inflammation. In the early phase of inflammation, these molecules are recognized as mediators that promote vasodilatation, attraction, and activation of immune cells [39,40]. However, the accumulation and overproduction of NO and PGE_2_ are indicative of chronic inflammation or cancer. It has been reported that different types of *S. horneri* ethanol extracts inhibit the overproduction of NO and PGE_2_ in LPS-stimulated macrophage RAW264.7 cells [34,42,43,44,45,46]. Moreover, treatment with ethanol extract of *S. horneri* was shown to decrease NO and PGE_2_ levels in fine dust-induced RAW264.7 cells [47]. However, there have been no reports on the anti-neuroinflammatory effects of *S. horneri* using microglia, including BV2 cells. BV2 cells are immortalized murine microglial cells, which are commonly used as a model for evaluating microglial activation and the secretion of inflammatory mediators related to various neurodegenerative disorders. Therefore, in our study, pretreatment with 70% EtOH extract and its CH_2_Cl_2_-, EtOAc-, *n*-BuOH-, and water-soluble fractions attenuated LPS-induced production of NO in BV2 cells (Figure 5). However, despite their remarkable inhibitory effects on NO production, all subsequent experiments were carried out only with *S. horneri* extract and its CH_2_Cl_2_- and water-soluble fractions, owing to their better yields. Our results indicated that treatment with *S. horneri* extract and its CH_2_Cl_2_- and water-soluble fractions also reduced LPS-induced production of PGE_2_ in BV2 cells (Figure 6). In addition, various types of *S. horneri* extracts have been reported to inhibit the production of cytokines, such as TNF-α and IL-6, in concanavalin A-induced rat splenocytes and LPS- or fine dust-induced RAW264.7 cells [47,48]. It is well-known that pro-inflammatory cytokines are important mediators of the inflammatory response. Cytokines, such as TNF-α and IL-6, are produced by the immune system following infection and are necessary for tissue repair. However, excessive production of TNF-α and IL-6 may lead to serious inflammatory disorders, including septic shock, rheumatoid arthritis, atherosclerosis, and cardiovascular diseases [39,40]. As demonstrated in Figure 6, treatment with *S. horneri* extract and its CH_2_Cl_2_- and water-soluble fractions attenuated the production of TNF-α and IL-6.

NO and PGE_2_ are synthesized by iNOS and COX-2, respectively, which are important inflammation-related enzymes. Abnormal upregulation of iNOS and COX-2 has been shown to lead to pathological conditions and aggravate the severity of inflammatory diseases [49]. Therefore, we investigated whether *S. horneri* extracts and their fractions inhibit the expression of iNOS and COX-2. Our results showed that treatment with *S. horneri* extract and its CH_2_Cl_2_- and water-soluble fractions suppressed the protein expression levels of iNOS and COX-2 (Figure 7). The induction of pro-inflammatory mediators and cytokines, including NO, PGE_2_, TNF-α, IL-6, iNOS, and COX-2, is modulated by transcription factors such as NF-κB, which is a major signaling molecule that activates various genes involved in the modulation of inflammation. Therefore, inhibition of NF-κB activation by phosphorylation and degradation of IκB-α, followed by NF-κB p65/p50 heterodimer translocation into the nucleus, may be an effective treatment strategy for inflammatory diseases. Recently, many plant-derived compounds have been investigated as potential inhibitors of NF-κB signaling. Moreover, many studies have been conducted on diverse compound classes, such as polyphenols, sesquiterpenes, diterpenes, triterpenes, and lignans [50]. In the present study, pretreatment with *S. horneri* extract and its CH_2_Cl_2_- and water-soluble fractions suppressed LPS-induced nuclear translocation of NF-κB (Figure 8A) and DNA-binding activity of p65 (Figure 8B). Moreover, immunofluorescence staining showed that pretreatment with this extract and its fractions blocked the translocation of p65 in LPS-induced BV2 cells (Figure 9). Taken together, our results demonstrate that the inhibitory effects of *S. horneri* extract and its fractions are involved in the inactivation of the NF-κB pathway.

Heme oxygenase (HO) is an important component of the cellular antioxidant system. As an HO derivative, HO-1 degrades intracellular heme to produce carbon monoxide (CO), iron, and biliverdin. Decomposed products such as CO, iron, and biliverdin, or HO-1 itself, are known to have anti-inflammatory and antioxidant effects, as well as prevent cell damage. HO-1 is induced by ultraviolet radiation, hydrogen peroxide, cytokines, hypoxia, and glutathione (GSH) consumption. Various types of cells can be considered part of the cell defense mechanism against stress. In particular, many studies have recently reported that HO-1 and its by-products might be able to inhibit neuronal cell damage caused by oxidative stress or inflammation [10,11,51]. The translocation of nuclear factor-E2-related factor 2 (Nrf2) into the nucleus is known to be most directly related to HO-1 regulation. Nrf2 is a redox-sensitive transcription factor that regulates the expression of various antioxidant enzymes, including HO-1. Nrf2 usually exists as an inactive complex with Keap1 in the cytoplasm. However, when activated, it moves into the nucleus and binds to the antioxidant response element site, thereby increasing the expression of antioxidant enzymes. It plays a central role in protecting various cells against oxidative stress by promoting the expression of genes and proteins of antioxidant enzymes [52,53]. In this study, among the extracts and their fractions, the CH_2_Cl_2_-soluble fraction showed the most remarkable HO-1 expression effects and increased Nrf2 expression in the nucleus (Figure 10). This is the first report on the biological action of *S. horneri* through HO-1 regulation in BV2 microglial cells. Because HO-1 and Nrf2 are well known to play a very important role in regulating oxidative damage and inflammation in most cell types [52,53], we investigated whether the antioxidant and anti-inflammatory effects of the CH_2_Cl_2_-soluble fraction were directly related to HO-1 expression in experiments pretreated with HO-1 inhibitors. Using this approach, we confirmed that the anti-oxidative and anti-neuroinflammatory effects of the CH_2_Cl_2_-soluble fraction were mediated by HO-1 and Nrf2 in BV2 cells (Figure 11). These results suggest that HO-1 mediates the effects of the CH_2_Cl_2_-soluble fraction of *S. horneri* and that these effects are regulated by the Nrf2 pathway. HT22 cells are mouse hippocampal cell line, which is immortalized and frequently used in oxidative stress models because they have no functional ionotropic glutamate receptors, causing glutamate-induced cell death [54,55]. Oxidative stress caused by glutamate induces cell death by increasing neuro-cytotoxicity or production of ROS [56,57]. In this experiment, it was confirmed that the CH_2_Cl_2_-soluble fraction had antioxidant activity that significantly decreased glutamate- stimulated oxidative stress and ROS production in HT22 cells, and that the properties were related to the expression of HO-1 (Figure 13). Therefore, when our results are summarized, it can be seen that 70% extract of *S. horneri* and especially the CH_2_Cl_2_-soluble fractions are valuable as a leading substance for the development of new drugs with inflammatory and antioxidant activities in neurodegenerative disorders. Further studies should be conducted to confirm the therapeutic effect of *S. horneri* 70% EtOH extract and its active compounds on neurodegenerative diseases using in vivo models.

## 5. Conclusions

In summary, among the extracts and their fractions, the CH_2_Cl_2_-soluble fraction showed the most remarkable scavenging activity in the DPPH and ABTS assays among the *S. horneri* extract and all fractions. *S. horneri* extract and its CH_2_Cl_2_- and water-soluble fractions exerted anti-neuroinflammatory effects by decreasing the production of inflammatory mediators, such as NO, PGE_2_, IL-6, and TNF-α, and the protein expression levels of iNOS and COX-2. These anti-neuroinflammatory properties were found to be related to NF-κB pathway inactivation in LPS-induced BV2 cells. In addition, the CH_2_Cl_2_-soluble fraction showed the most remarkable HO-1 expression and increased Nrf2 expression in the nuclei of BV2 cells. Moreover, it was also confirmed that CH_2_Cl_2_-soluble fractions have neuroprotective effect related to the expression of HO-1 by reducing glutamate-induced oxidative stress and ROS production in HT22 cells. Taken together, our findings suggest that marine natural products, such as CH_2_Cl_2_-soluble fractions of *S. horneri*, can attenuate oxidative action and neuroinflammatory responses via regulation of HO-1 signaling, demonstrating their potential in the treatment of neuroinflammatory diseases.

## Figures and Tables

**Table 1:** Proximate composition of *S. horneri*.

Proximate Composition (% Dry wt.)				
Moisture	Carbohydrate	Ash	Crude Fat	Crude Protein
14.19 ± 0.39	45.99 ± 1.61	20.93 ± 0.41	4.37 ± 0.14	14.52 ± 1.06

Data are shown as the mean ± SEM values (*n* = 3). Carbohydrate content (%) = 100 − (% moisture + % protein + % lipid + % ash).

**Table 2:** The monosaccharide composition analysis of 70% EtOH extract of *S. horneri* and its CH_2_Cl_2_- and water-soluble fractions. n.a.*: not applicable peaks.

No.	Name	70% EtOH Extract		CH_2_Cl_2_-Soluble Fractions		Water-Soluble Fractions	
		Content [%]	Amount [mg/mg]	Content [%]	Amount [mg/mg]	Content [%]	Amount [mg/mg]
1	Arabinose	2.800	-	0.540	-	3.700	-
2	Fucose	12.940	0.002	9.350	0.004	21.610	0.001
3	Galactose	56.750	0.007	82.820	0.033	20.400	0.001
4	Glucose	13.690	0.002	-	-	32.990	0.002
5	Rhamnose	1.270	-	0.610	-	2.980	-
6	n.a.*	12.550	0.002	6.680	-	18.330	-

## Data Availability

The data presented in this study are available within the article. Other data that support the findings of this study are available upon request from the corresponding author.
